# Identification of differentially expressed microRNAs in metastatic melanoma using next-generation sequencing technology

**DOI:** 10.3892/ijmm.2014.1668

**Published:** 2014-02-25

**Authors:** MIN QI, XIAOYUAN HUANG, LEI ZHOU, JIANGLIN ZHANG

**Affiliations:** 1 Department of Plastic Surgery, Xiangya Hospital, Central South University, Changsha, Hunan 410008, P.R. China; 2 Department of Dermatology, Xiangya Hospital, Central South University, Changsha, Hunan 410008, P.R. China

**Keywords:** metastatic melanoma, differentially expressed microRNAs, target gene prediction, interaction network, enrichment analysis

## Abstract

In this study, we investigated differentially expressed microRNAs (miRNAs or miRs) and their functions in metastatic melanoma using next-generation sequencing technology. The GSE36236 data set was downloaded from the Gene Expression Omnibus (GEO) database and 4 primary cutaneous melanoma samples (used as controls) and 3 metastatic melanoma samples were selected from 31 samples for further analysis. Firstly, the differentially expressed miRNAs were screened by limma package in R language. Secondly, the target genes of the miRNAs were retrieved with TargetScanHuman 6.2, and the interactions among these genes were identified by String and an interaction network was established. Finally, functional and pathway analyses were performed for the genes in the network using Expression Analysis Systematic Explorer (EASE). A total of 4 differentially expressed miRNAs (hsa-miR-146, hsa-miR-27, hsa-miR-877 and hsa-miR-186) were obtained between the metastatic melanoma and primary cutaneous melanoma samples. We predicted 101 high-confidence target genes of hsa-miR-27 and obtained a network with 41 interactions. Finally, functional and pathway analyses revealed that the genes in the network were significantly enriched at the transcriptional level. Differentially expressed miRNAs were identified in the metastatic melanoma compared with the primary cutaneous melanoma samples and the target genes of hsa-miR-27 were found to be significantly enriched at the transcriptional level. The results presented in our study may prove helpful in the diagnosis and treatment of metastatic melanoma.

## Introduction

The incidence of metastatic melanoma and mortality rate has continually increased over the past decades (1,2). The invasive and metastatic ability are the most fundamental features of cancer cells which differ from normal cells, eventually leading to tumor recurrence, progression and ultimately, death (3). The median survival of patients with metastatic melanoma is <1 year (4). Thus, an improvement in therapy for metastatic melanoma is mandatory.

A number of studies have revealed that microRNAs (miRNAs or miRs) are associated with the metastasis of colon cancer, breast cancer and lung cancer cells (5–7). Over the past few years, the association between miRNAs and tumors has become a research hotspot. Certain miRNAs have been proven to be potential biomarkers in clinical diagnosis (8,9), which have the most extensive gene regulation functions on several levels (10) and are involved in a series of important biological processes, including early embryonic development, cell proliferation, apoptosis, cell death and fat metabolism, and even the differentiation of stem cells (11). Due to the advances in miRNA research, an increasing number of studies have demonstrated that miRNAs regulate important tumor-related genes, and are thus closely related to the development and occurrence of tumors. For example, miR-7 and miR-331–3p have been shown to regulate the expression of epidermal growth factor receptor (EGFR) and human EGFR, which are biomarkers of human tumors (12). Jiang *et al* demonstrated that miRNA-155 acts as a suppressor of the cytokine signaling 1 gene that is a tumor suppressor in breast cancer (13). However, a number of tumor-associated miRNAs remain unexploited, and the mechanisms responsible for the regulatory functions of miRNAs in metastatic melanoma are not yet fully understood. Therefore, miRNAs may provide a novel treatment method for cancers.

Given the detrimental effects of metastatic tumors and the growing development of sequencing technology, small RNA sequencing was adopted in our study to identify the differentially expressed miRNAs in metastatic melanoma compared with primary cutaneous melanoma samples. The target genes were predicted, and functional and pathway analyses were performed to reveal their roles in the progression of metastasis. Our findings may provide new insight into the diagnosis and treatment of metastatic tumors.

## Materials and methods

### Sequencing data

The GSE3623 sequencing data, including a total of 31 samples, was downloaded from the Gene Expression Omnibus (GEO) database. A total of 7 melanoma samples were selected, including 4 primary cutaneous melanoma samples (used as controls) and 3 metastatic melanoma samples. Raw data were obtained in SRA format, as previously described (14). The platform used was GPL9052 Illumina Genome Analyzer (*Homo sapiens*). Deep sequencing was performed on various tissues and sequencing data were obtained. We transformed the data from SRA format to FASTQ format, as previously described (15) using the SRA Toolkit (16). Sequence alignments were then carried out with Bowtie alignments (17), allowing as many as 2 mismatches. Bowtie is a rapid and memory-saving tool for short sequence assembly. It aligns short sequences to reference genomes and can read base pairs as long as 1024 bp, which renders it extremely suitable for next-generation sequencing technologies (18). The expression level of miRNAs was determined using miRDeep, as previously described (19), which has been proven to be of high sensitivity and accuracy (20).

### Screening of differentially expressed miRNAs

After the expression levels were determined, the differentially expressed miRNAs were screened out between the metastatic melanoma and primary cutaneous melanoma (controls) samples with limma package in R language (21); a p-value <0.05 and |logFC|>1 were set as the cut-off values.

### Prediction of target genes of miRNAs

Target genes were predicted using TargetScanHuman 6.2, which is a software package developed by Lewis *et al* (22). This software package was used for predicting target genes in mammalian species based on conservations across species and thermodynamic features of miRNA-target gene complexes (23). Target genes with a prediction score >0.9 were regarded as of high confidence.

### Interaction network construction of target genes

Genes usually interact with each other to exert certain biological functions (24). Therefore, genes which interact with target genes were identified by the String database (25) and the interaction network of these genes was then established to broaden the understanding of their functions. The String database can rate the interactions from the aspects of homology, experiment and text mining.

### Functional and pathway analyses

Functional and pathway analyses were performed for the genes in the network with Expressing Analysis Systematic Explorer (EASE) (26) (p<0.05) which can identify the significant Gene Ontology (GO) terms and the genes that exist in the GO categories (27).

## Results

### Sequencing data

After the raw data were transformed into FASTQ format, the sequence read length was 36 bp. The number of original reads, miRNA alignments and miRNA types for each sample are listed in Table I. The expression levels of the miRNAs in each sample were then determined (data not shown).

### Differentially expressed miRNAs

A total of 4 differentially expressed miRNAs were identified in the metastatic melanoma samples as compared with the primary cutaneous melanoma (control) samples according to the criteria (p<0.05 and |logFC|>1). These 4 miRNAs were hsa-miR-146, hsa-miR-27, hsa-miR-877 and hsa-miR-186. The information for these 4 miRNAs is presented in Table II.

### Target genes and the interaction network

**Figure 1:**
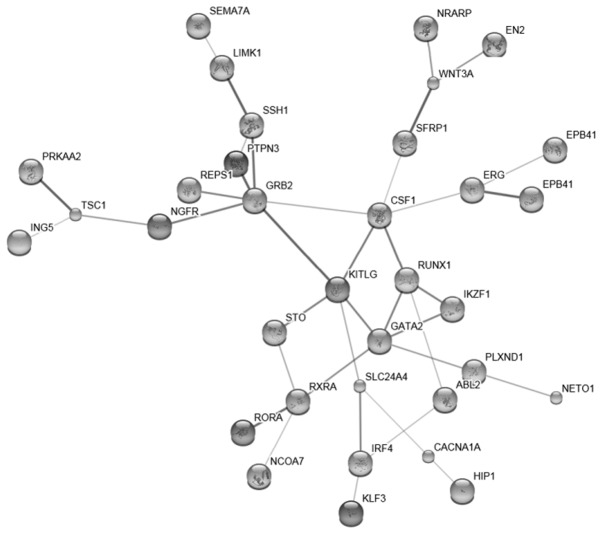
Interaction network of the target genes. A total of 101 target genes of has-miR-27 and 41 interactions were obtained. We then constructed a network based on these interactions.

A total of 101 target genes with high confidence were found for hsa-miR-27 (data not shown). A total of 41 interactions (data not shown) were disclosed and the network was then constructed (Fig. 1).

### Functional and pathway analyses of genes in the network

**Figure 2:**
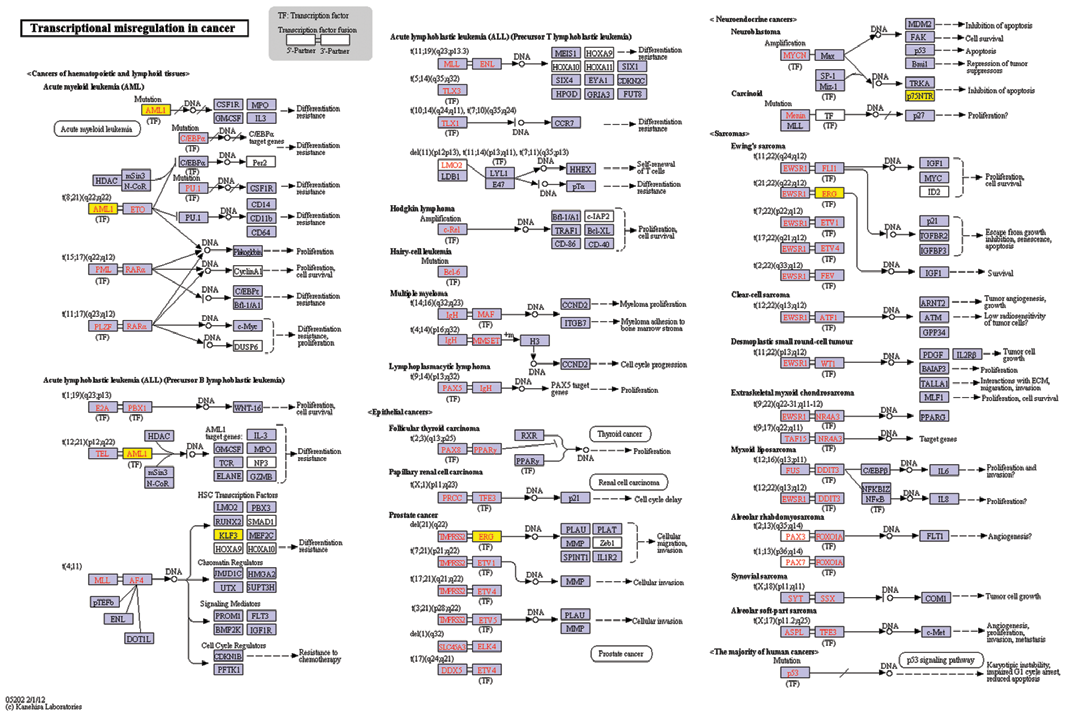
Pathways associated with enriched genes in the network. Genes in the yellow boxes are the target genes of differentially expressed hsa-miR-27.

A total of 4 significant functional terms and 1 pathway were identified (Table III) for the genes in the interaction network. The most significant function was transcription, while the pathway of transcriptional misregulation in cancer was also revealed (Fig. 2). The genes which were enriched at the transcriptional level, included inhibitor of growth family, member 5 (ING5), GATA binding protein 2 (GATA2) and erythroblast transformation-specific (ETS)-related gene (ERG). Other genes, such as Ikaros family zinc finger 1 (IKZF1), interferon regulatory factor 4 (IRF4) and runt-related transcription factor (RUNX), as well as retinoid X receptor, alpha (RXRA) and RAR-related orphan receptor A (RORA) were also included in the list (Table II).

## Discussion

We identified 4 differentially expressed miRNAs by next-generation sequencing technology. In order to determine the functions of these miRNAs, the target genes of hsa-miR-27 were predicted and the interaction network of the genes was constructed. Finally, the functions of and pathways associated with these genes were analyzed in order to further elucidate the mechanisms responsible for metastatic melanoma.

The 4 differentially expressed miRNAs were hsa-miR-146, hsa-miR-27, hsa-miR-877 and hsa-miR-186. Rabinowits *et al* found the overexpression of hsa-miR-146 in lung cancer (28). Katakowski *et al* demonstrated that the expression of miR-146b-5p inversely correlated with glioma invasiveness in the brain (29). The study by Mertens-Talcott *et al* indicated that miR-27a is overexpressed in breast cancer cells, and that cell proliferation is decreased by the inhibition of this miRNA using antisense molecules in MDA-MB-231 cells (30). Scott *et al* reported that in SKBr3 breast cancer cells, the inhibitor of histone deacetylases, LAQ824, rapidly decreased miR-27a levels (31). miR-27a has also been shown to be upregulated in renal cell carcinoma as compared with normal kidneys (32) and acts as an oncogene in gastric adenocarcinoma (33). Guttilla and White further indicated that in breast cancer cells, miR-27a regulated the expression of FOXO1 (34), a putative tumor suppressor. The overexpression of miR-877 has been reported in endometrial serous adenocarcinomas (35). In a previous study, when glyceollins were applied for the treatment of triple-negative breast cancer, the downregulation of miR-877 was observed (36). Baffa *et al* indicated that hsa-miR-186 was differentially expressed in paired primary and metastatic cancers (37). Leidinger *et al* demonstrated that hsa-miR-186 combined with other miRNAs was able to distinguish melanoma patients from healthy individuals (38). Therefore, we consider that these 4 miRNAs are potential targets for modulating the metastasis of melanoma cells.

To further elucidate the regulatory mechanisms, the target genes of the 4 miRNAs were predicted by TargetScan. A total of 101 target genes were revealed for hsa-miR-27. Functional enrichment analysis revealed that the most significantly enriched term was transcription, indicating that a number of gene expressions are regulated, which may lead to the acquisition of metastatic tumor cells. In addition, several target genes have been disclosed and are worthy of further investigation. ING5 is predicted to be one of the target genes which is linked to tumorigenesis in human head and neck carcinoma (39). GATA2 is a member of the GATA family of zinc-finger transcription factors. Bohm *et al* found that the high expression of GATA2 is associated with metastatic progression and biochemical recurrence in prostate cancer through the regulation of key androgen-regulated genes (40). ERG is a member of the ETS family and a number of studies have investigated its role in prostate cancer (41,42).

Overall, next-generation sequencing technology was adopted in this study to identify the differentially expressed miRNAs in metastatic melanoma as compared with primary cutaneous melanoma samples. Moreover, the functions and pathways of target genes were revealed, which may provide insight into the regulatory mechanism. The data presented in our study may prove beneficial in the diagnosis and treatment of metastatic melanoma and may aid in the development of novel clinical applications using miRNAs. However, further sutdies are required to confirm our results.

## Figures and Tables

**Table I:** Statistical information of the sequencing data.

Group	Accession no.	No. of original reads	No. of miRNA alignments	No. of miRNA types
Metastatic melanoma	GSM893568	402556	17847	259
	GSM893576	1226137	18005	123
	GSM893577	708653	16493	262
	GSM893578	395686	31687	279
Primary melanoma	GSM893564	97403	85049	338
	GSM893566	464804	40816	251
	GSM893573	1279059	40267	

**Table II:** Details for the 4 differentially expressed miRNAs.

ID	p-value	|logFC|
hsa-miR-146	0.005384	1.91716394
hsa-miR-27	0.035685	1.49264032
hsa-miR-877	0.039706	2.73696559
hsa-miR-186	0.041463	1.1356551

**Table III:** Functional and pathway analyses of the genes in the network.

Term - pathway	p-value	Genes
GO:0006350 - Transcription	0.001954	ING5, GATA2, ERG, IKZF1, RXRA, NCOA7, IRF4, RORA, RUNX1, HIP1, KLF3
GO:0045449 - Regulation of transcription	0.003148	ING5, GATA2, ERG, IKZF1, RXRA, NCOA7, IRF4, EN2, RORA, RUNX1, HIP1, KLF3
GO:0043228 - Non-membrane bounded organelle	0.016522	GATA2, SSH1, PTPN3, EPB41, TSC1, IKZF1, EPB41L4A, KITLG, IRF4, FGD6, ABL2, HIP1
GO:0043232 - Intracellular non-membrane bounded organelle	0.016522	GATA2, SSH1, PTPN3, EPB41, TSC1, IKZF1, EPB41L4A, KITLG, IRF4, FGD6, ABL2, HIP1
KO:05202 - Transcriptional misregulation in cancer	0.000498	ERG, KLF3, AML1
